# Recent changes in the upper oceanic water masses over the Indian Ocean using Argo data

**DOI:** 10.1038/s41598-023-47658-9

**Published:** 2023-11-20

**Authors:** Abhijit Shee, Sourav Sil, Avijit Gangopadhyay

**Affiliations:** 1 Ocean Analysis and Modelling Laboratory, School of Earth, Ocean and Climate Sciences, Indian Institute of Technology Bhubaneswar, Bhubaneswar, Odisha India; 2 Centre for Atmospheric and Oceanic Sciences, Indian Institute of Science, Bengaluru, Karnataka India; 3 School for Marine Science and Technology, University of Massachusetts at Dartmouth, Dartmouth, MA USA

**Keywords:** Physical oceanography, Physical oceanography

## Abstract

Utilizing Argo data from 2003 to 2019, we examine thermohaline changes in the Indian Ocean within the upper 700 m. Widespread warming is observed except in the Southern Indian Ocean. Increasing salinity is obtained over all regions except the Bay of Bengal and Southern Indian Ocean. Thermohaline trends in regional water masses at various depths are first decomposed into spice and heave components, and then linked to processes like pure heave, pure freshening and pure warming. Three consistent patterns across all seven regions are: (1) Below 300 m spice dominates heave; (2) The freshening process within the spice component is the primary driver below 300 m; (3) Spice primarily influences salinity changes along isobars. The warming of Arabian Sea’s Subsurface Minima and the Indian Equatorial Water are primarily dictated by spice and heave, respectively. Freshening of the Bay of Bengal Water is linked to heave changes under pure freshening and pure heave processes. In the upper 250 m of the western equatorial, southern Indian Ocean, and Seychelles–Chagos Thermocline Ridge, salinity rises due to spice under pure freshening. The southern Indian Ocean’s advected mode water shows freshening and cooling trends due to pure freshening.

## Introduction

Ocean temperature and salinity directly influence Earth’s atmosphere and are pivotal in global climate change. The Indian Ocean (IO) differs from the other two tropical oceans due to its northern landlocked nature and unique basin dynamics. The tropical IO belongs to the Indo-Pacific warm pool and hosts multiple climate oscillation zones^1^. As a crucial part of the global ocean conveyer belt^2^, this ocean significantly affects the global climate. The Bay of Bengal (BoB) in the northeastern part of the IO, is warm and less saline, whereas the Arabian Sea (AS) in the northwestern part of the IO contains colder and more saline waters. Both basins significantly influence regional weather patterns, like the Indian monsoons and tropical cyclones. Due to its weak and variable winds, the equatorial region is well known for Ekman suction, or upwelling^3^. The Seychelles–Chagos Thermocline Ridge (SCTR, 5° S–12°S, 55° E–90°E), in the tropical south IO is an open-ocean upwelling area, recognized for thermocline uplift and pronounced air-sea interaction, resulting from cyclonic wind stress curl^4–6^. The tropical IO links to waters in south of 30° S via two vertical overturning cells: one, a shallow subtropical cell with upwelling in the SCTR, and the other, a cross-equatorial cell upwelling in the AS’s western region^7^.

Earlier studies^8–12^ have observed a warming trend in the IO over the past 50 years, using satellite data and model reanalysis datasets. Since 2003, the IO’s warming trend has contributed to nearly 70% of the global ocean heat content (OHC) trend^13–15^. Measuring changes in OHC is essential for studying cyclogenesis^16^, monsoons^17^, and other air-sea interactions. Likewise, salinity and freshwater changes in the Indian and Arctic Oceans, particularly in terms of the ocean freshwater content (OFC) have been investigated^18–20^. In the IO, the BoB is the primary source of freshwater due to significant precipitation and river runoff, influencing the AS and regions south of the equator^18^. Changes in IO’s OHC also impact marine heatwaves, oxygen distributions, ecosystems, and the economies of surrounding countries^21,22^.

The study of ocean heat and freshwater content (OHC and OFC) often involves integrated measures of the heat and freshwater within the top 50–200 m. This range typically encompasses the wind-driven mixed layer, the cyclone-induced upper layers or extends down to a selected isotherm^23^ or depth^24^. A primary aim of our research is to explore and document the heat and freshwater content and their trends up to 700 m deep in the water column. Within this depth, various studies have identified distinct and significant water masses. Refer to Table 1 for a list of these water masses and their respective references. We chose a depth 700 m for two reasons: (1) Many observational studies before the Argo era focused only up to this depth^25^, enabling us to juxtapose our recent findings with earlier trends; and (2) Some current studies on global oceans have concentrated on the OHC within the top 700 m over the past two decades^15,26^ allowing for comparisons with contemporary trends. Utilizing Argo data from 2003 to 2019, we documented these OHC and OFC trends across seven regions for the top 700 m. Additionally, we linked these trends to various warming and freshening mechanisms, drawing on the water-mass change analysis suggested by Bindoff and Mcdougall^27^. Their model assesses temperature and salinity changes in the water column and interprets the role of changes in atmospheric forcings through pure warming, pure freshening and pure heave processes. Similar evaluations have been conducted in numerous studies for the Global Ocean^13,28,29^, Southern Hemisphere oceans^30^, and regions of the Pacific Ocean^27,31–33^, Atlantic Ocean^34–41^, Indian Ocean^42,43^ and Southern Ocean^44^. Yet, the tropical regions in the IO remain largely uncharted in terms of water-mass alterations. While satellites have informed the study of interannual variability at the ocean’s surface, subsurface observations were constrained by the lack of vertical profiles. The introduction of Argo data has transformed our ability to estimate the seasonal to decadal variability of water mass changes in the upper 2000 m in the ocean on a regional scale. In the IO, comprehensive Argo data has been available since 2003.

**Table 1:** Details of the water masses observed in the upper layers of the Indian Ocean regions.

Water mass	Region and Depth (m)	${\sigma }_{t}$(kg/m3)	Salinity (psu)	Temperature ($\mathrm{^\circ{\rm C} }$)	A few relevant References
Arabian Sea high salinity water	AS (0–100 m), WEIO (0–80 m)	22.8–24.5	35.3–36.7	24–29	Shaji and Gangopadhyay^53^
Arabian sea subsurface salinity minimum water	AS (125–200 m)	25.5–26.5	35.1–36.2	16–19	
Persian Gulf water	AS (200–400 m)	26.2–26.8	35.1–37.9	13–19	
Red Sea water	AS (below 500 m)	27–27.4	35.1–35.6	9–11	
Bay of Bengal water	BoB (0–50 m), EEIO (0–50 m)	21–23	25–35	24.2–29.7	Sengupta et al.^54^
Mixing zone	BoB (50–150 m)	22–25.2	34.4–34.9	17–28.3	
Indonesian throughflow	BoB (200–500 m)	26.5–27	34.9–35.1	9.6–15.3	
Indian Ocean deep water	BoB (below 500 m)	27–28.5	34.7–35	3–9.4	
Equatorial surface water	CEIO (0–100 m), EEIO (50–100 m)	22–23.8	33.5–36	25–29.5	Emery^46^
Indian equatorial water	WEIO, CEIO, EEIO and SCTR (100–500 m)	23.5–27	34.6–35.8	8–23	
Red Sea-Persian Gulf intermediate Water	WEIO, CEIO, EEIO and SCTR (below 580 m)	26–27.5	34.8–35.4	5–14	
Indonesian upper water	SIO (0–150 m)	< 24.9	> 23	34.4–35.5	Harms et al.^55^
Subtropical surface water	SIO (150–350 m)	24.9–26.4	12–22	35.1–35.8	
Subantarctic mode water	SIO (below 350 m)	26.4–26.9	6–14	34.5–35.5	

**Figure 1:**
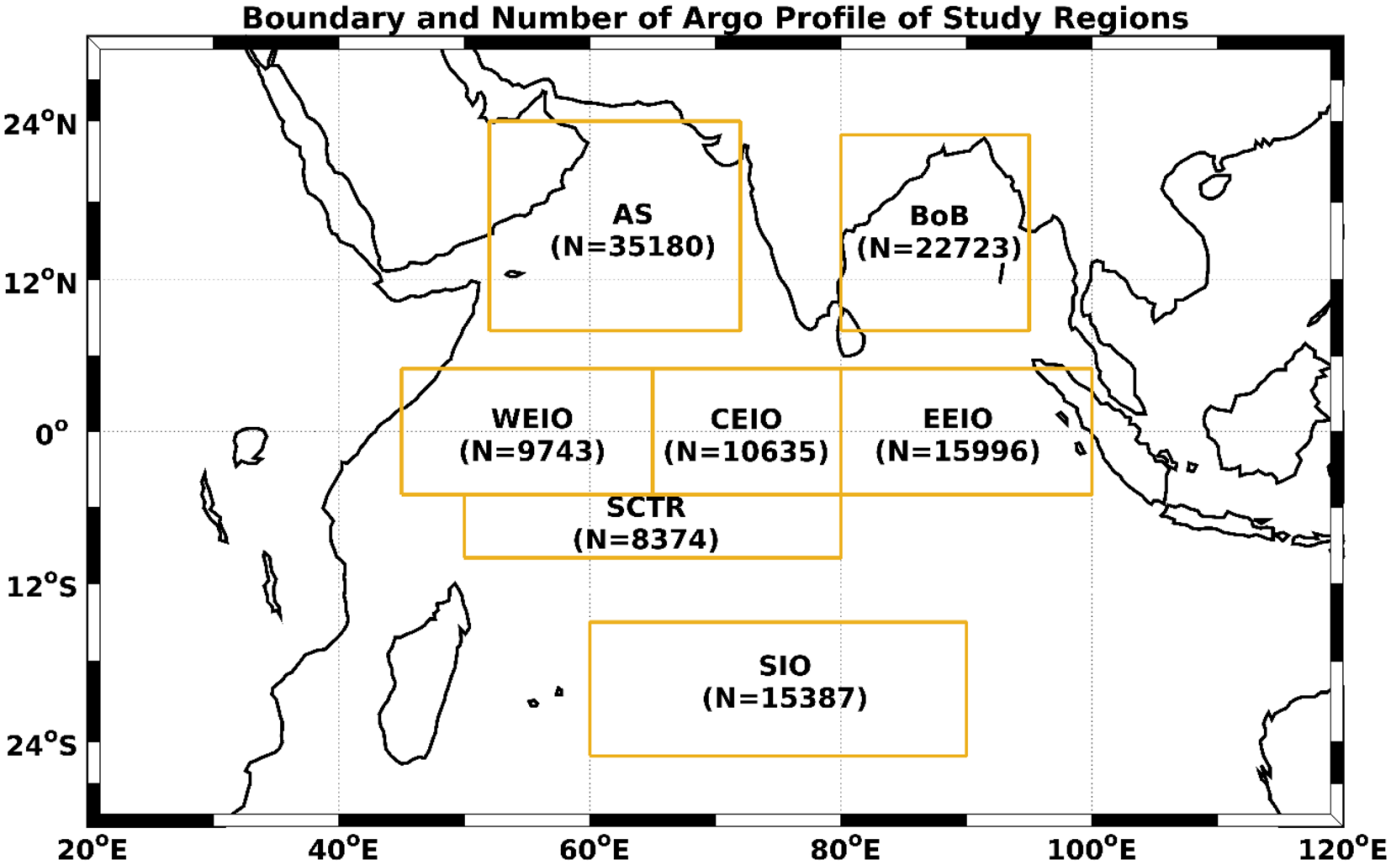
The boundaries of the seven different study regions within the IO are marked by boxes. In this study, the abbreviations AS, BoB, WEIO, CEIO, EEIO, SCTR and SIO correspond to the Arabian Sea, Bay of Bengal, western equatorial IO, central equatorial IO, eastern equatorial IO, Seychelles–Chagos Thermocline Ridge and southern IO, respectively. The total count of Argo profiles from 2003 to 2019 is displayed for each region. It’s important to note that every region had a minimum of twenty profiles monthly since 2005. During 2003 and 2004, there were a at least four profiles monthly (typically two different Argos).

Interannual variability in OHC and OFC correlates closely with changes in ocean water masses^45^. The IO’s unique geographic conditions give rise to a complex structure of water masses in its upper layer^46^. Helland-Hansen^47^ proposed that water masses can be identified by plotting temperature (T) against salinity (S). Deviations from the typical T–S curve can be attributed to the intrusion of foreign water masses originating from different regions. In our study, an annual mean climatology T–S representation using Argo temperature and salinity profiles from 2003 to 2019 for each of the seven regions, which will be detailed subsequently and are illustrated in Fig. 1.

Given the unique geography of the tropical IO and its susceptibility to various processes affecting water-mass variations, we focused on seven distinct regions in this study, as shown in Fig. 1: Arabian Sea (AS: 52° E–72° E, 8° N–24° N), Bay of Bengal (BoB: 80° E–95° E, 8° N–23° N), West Equatorial IO (WEIO: 45° E–65° E, 5° S–5° N), Central Equatorial IO (CEIO: 65° E–80° E, 5° S–5° N) and East Equatorial IO (EEIO: 80° E–100° E, 5° S–5° N), Seychelles–Chagos Thermocline Ridge (SCTR: 50° E–80° E, 10° S–5° S) and Southern IO (SIO: 60° E–90° E, 25° S–15° S). Initially, we verified the accuracy by comparing near surface temperature (NST) and salinity (NSS) from quality-controlled Argo data with satellite-derived sea surface temperature (SST) and salinity (SSS). Following this, we examined temperature and salinity changes along isobars, decomposing these into variations in spice and heave for each of the seven regions. Finally, these decomposition results were analyzed in the context of pure warming, pure freshening and pure heave processes.

## Results

### Comparison of satellite and argo data

**Figure 2:**
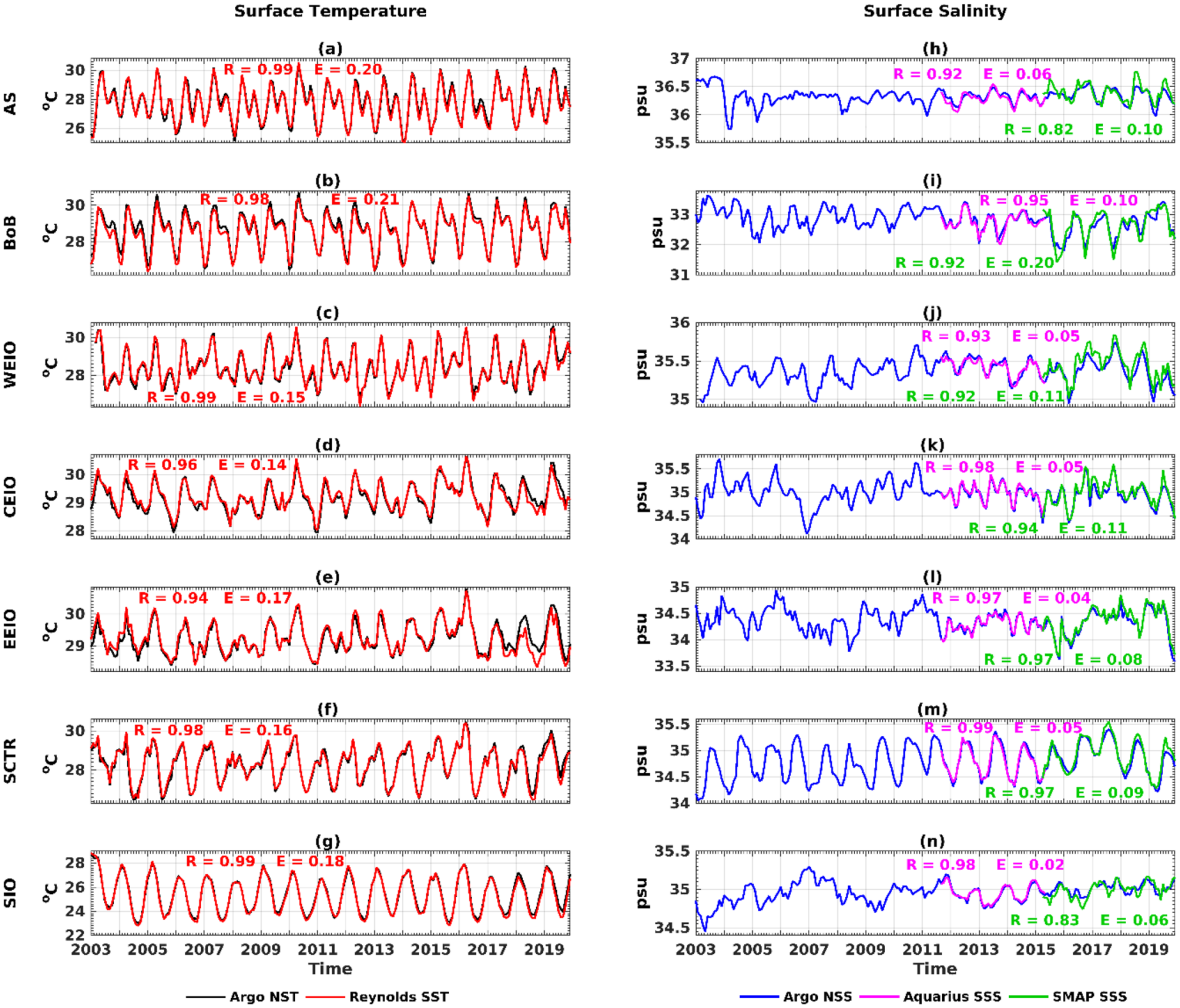
The left column represents the time series of OISST (red line) and Argo temperature at 5 m depth (black line), and the right column represents the time series of Aquarius SSS (magenta line), SMAP SSS (green line) and Argo salinity at 5 m depth (blue line) during 2003–2019 for the respective regions. R and E denote the correlation coefficient and root mean square error between Argo and satellite in that region. The colors of R and E indicate the satellite products with which Argo data are correlated.

Figure 2 displays the temporal evolutions of monthly averaged Argo-derived NST and NSS. These are juxtaposed with satellite-retrieved SST (Fig. 2a–g) and SSS (Fig. 2h–n). Over a 17 year time span (2003–2019), the Argo NST and Optimum Interpolation Sea Surface Temperature (OISST) demonstrate a strong correlation (r $\ge$ 0.94) across all regions. Additionally, Argo-derived NSS shows a high correlation (r $\ge$ 0.92) with the available Aquarius SSS from September 2011 to May 2015 in every region (Fig. 2). Furthermore, the Argo NSS aligns well with the SSS data from the Soil Moisture Active Passive (SMAP) satellite product (refer to the data section) during the data-available period of April 2015 to December 2019. The correlation values are approximately 0.82 for AS and SIO, while they are $\ge$ 0.92 for the other regions (Fig. 2). The error, measured as the root mean square difference, is consistent in their overlapping periods across all areas. However, an exception arises in the BoB, where the SMAP SSS value is notably lower than the Argo NSS.

### Observed thermohaline trends

**Figure 3:**
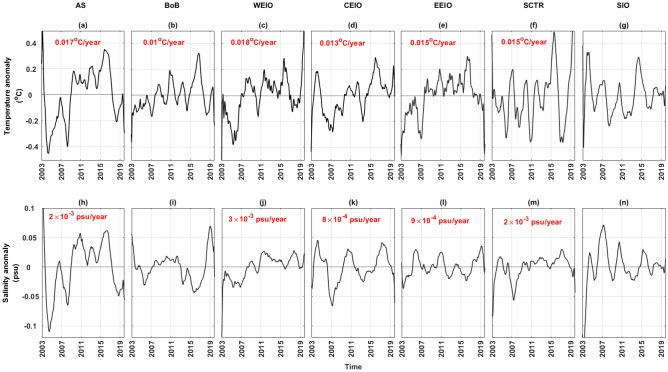
Time series of temperature (top rows) and salinity (bottom rows) anomalies averaged over the upper layer (0–700 m) from Argo for the seven regions. Significant (*p*-value $\le$ 0.1) trend values for each region are indicated in red. The plots are smoothed with a 13-point moving average function.

While the NST and NSS capture surface signals largely influenced by atmospheric forcings, ocean heat and freshwater contents provide insight into the interior processes driving both intra- and interannual variations. Heat content changes significantly influence sea level rise due to thermal expansion and air-sea interactions. Conversely, changes in freshwater content play a crucial role in subsurface heat trapping and anomalous temperature responses^48,49^. In this study, we emphasize depth-averaged (0–700 m) temperature and salinity rather than the upper ocean heat and freshwater content. The temperature anomaly demonstrates warming trends across all regions, except the SIO (Fig. 3), with notably high values in the western equatorial Indian Ocean and subdued values in the BoB. Similarly, an increasing saltening trend is evident in the salinity anomaly for all areas, barring the BoB and southern Indian Ocean (Fig. 3). This trend showcases higher values in the eastern equatorial Indian Ocean and milder ones in the AS and SCTR regions. Conversely, the near-surface temperature anomaly reveals significant positive trends in both central and eastern equatorial Indian Ocean regions. The near-surface salinity displays a significant negative trend in the BoB and positive trends in the western equatorial IO, SCTR and SIO regions. The thermohaline trends detailed in Fig. 3, derived from Argo data, align closely with recent studies^50,51^.

### Observed Trends along Isobars and their decomposition

**Figure 4:**
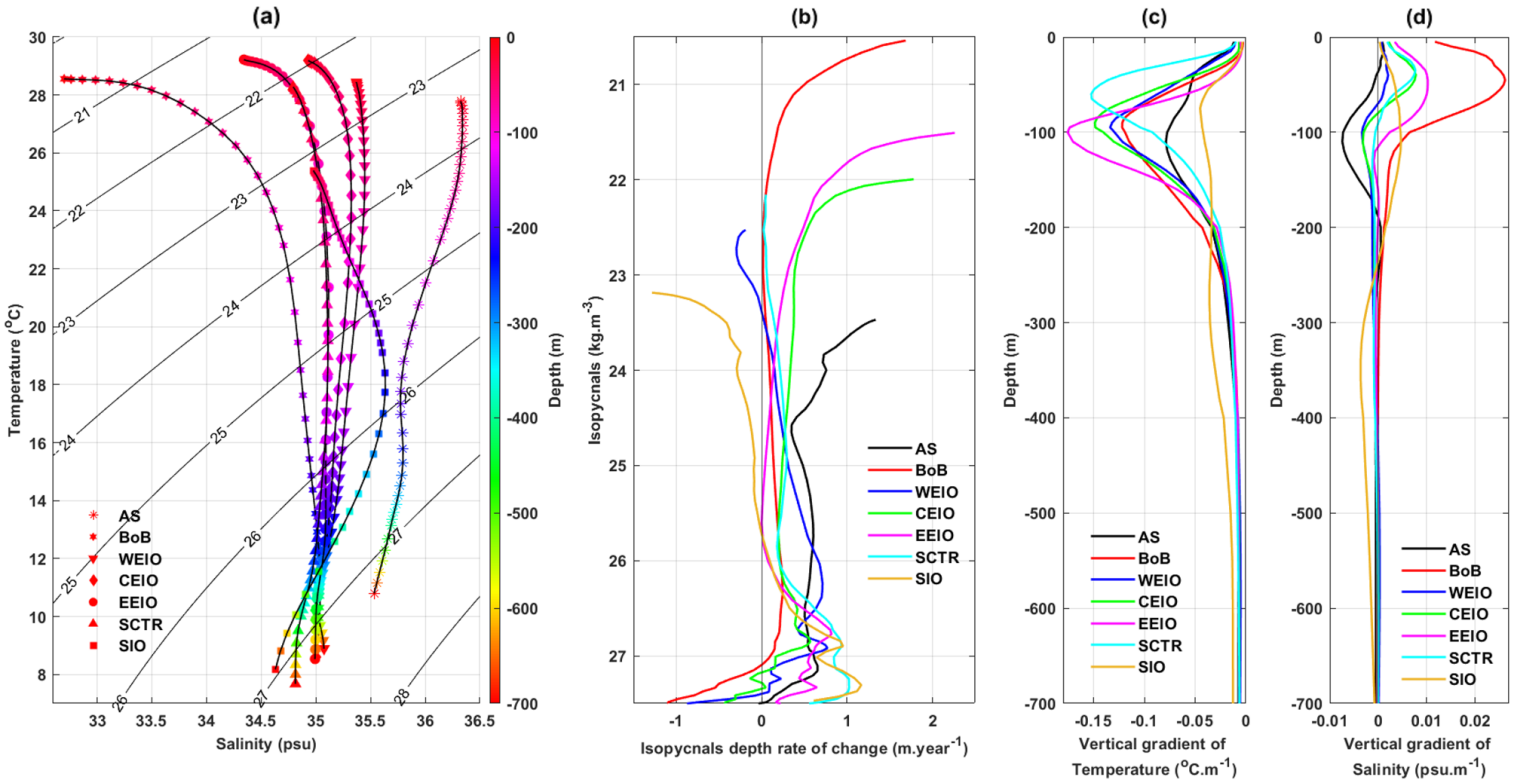
(**a**) Annual average Temperature–salinity (T–S) diagram based on Argo (2003–2019) data in the upper ocean (0–700 m) for seven regions. Note that EEIO and SCTR waters are almost indistinguishable in this annual representation. The color bar has the depth range for the respective water masses. (**b**) Time rate of change in isopycnal depth over 2003–2019 for seven regions. Vertical gradient of mean (**c**) temperature and (**d**) salinity over the period of 2003–2019 for different regions. Here in gradient estimation, depth is considered positive into the ocean. Thus, negative vertical gradient implies decreasing of the properties with depth.

Pronounced variations in depth-averaged upper oceanic temperature and salinity as seen in Fig. 3, highlight the lateral and vertical redistribution of temperature and salinity over time. Therefore, gaining a comprehensive understanding of these upper oceanic thermohaline changes, linked to the water masses depicted in Fig. 4a, is crucial. Within the IO’s upper ocean regions, vertical temperature gradients are consistently negative with increasing depth, signifying warm water layered over cold water (Fig. 4c). Yet, the vertical salinity gradient is not uniformly positive across all regions, suggesting layers of fresher water atop saltier layers (Fig. 4d). A region’s isopycnals relate to water masses, which can originate elsewhere, and can be identified by plotting temperature (T) against salinity (S)^52^. Comprehensive descriptions of these upper oceanic water masses in the IO regions are presented in Fig. 4a and detailed in Table 1.

**Figure 5:**
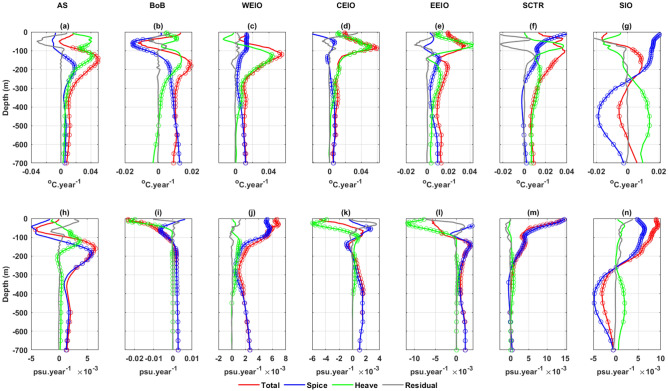
The total trend (red) decomposed into spice (blue) and heave (green) components for the upper 700 m in temperature (top rows) and salinity (bottom rows) profiles in the seven IO regions. The gray line represents the residual term of decomposition. Circles on the plots indicate the trends that have passed the 90% significance test (*p*-value $\le$ 0.1) and rejected the null hypothesis.

To delve deeper into the climate change effects on the water masses, it is essential to segregate isobaric temperature and salinity changes into their spice and heave components as visualized in Fig. 5. According to Eq. (3), the heave component arises from the movement of isopycnals combined with vertical temperature or salinity gradients. In our analysis, the heave component noticeably influences the upper 400 m temperature changes across all regions (Fig. 5). The impact of spice on isobaric salinity changes is more pronounced than that of the heave component in every region (Fig. 5). However, within the top 100 m of the BoB and both central and eastern equatorial regions, the heave component predominately affects isobaric salinity change. The residual, defined as the discrepancy between the total change and the combined effects of spice and heave, exhibits significant trends for both temperature and salinity changes in the upper 200 m (Fig. 5). This decomposition becomes intricate in these depths due to factors like air-sea interactions and mixing due to large vertical gradients^28,42^.

In the Arabian Sea, the deepening of isopycnals as depicted in Fig. 4b, corresponds with the presence of several water masses: the Arabian Sea High Salinity Water (0–100 m), Arabian Sea Subsurface Salinity Minimum (125–200 m), Persian Gulf Water (200–400 m) and Red Sea Water (below 500 m) as illustrated in Fig. 4a and detailed in Table 1^53,56–58^. Throughout this region, we observe negative vertical temperature gradients with a pronounced peak around 110 m, suggesting layers of warm waters over colder ones (Fig. 4c). Consequently, the heave component of temperature changes reveals a consistent warming trend, peaking at approximately 110 m (Fig. 5a). The spice component, in contrast, displays significant warming trends primarily below 100 m, with its apex around 180 m (Fig. 5a). The cumulative influence of these components results in overall temperature changes that exhibit warming trends along isobars, reaching its peak at roughly 150 m (Fig. 5a).

Regarding salinity, negative vertical salinity gradients appear between 50 and 180 m, peaking at 110 m. This pattern suggests the sandwiching of a low-salinity layer between the more saline waters of the Arabian Sea and Persian Gulf (Fig. 4d). As a result, the heave component of salinity changes indicates a pronounced saltening trend between 80 and 200 m, peaking again around 110 m (Fig. 5h). The spice component present dual trends: freshening above 100 m (peak at ~ 50 m) and saltening below (peak at ~ 180 m) (Fig. 5h). Salinity changes along isobars closely mirror the spice component, with the most significant saltening trend found at about 150 m depth (Fig. 5h). Overall, our analysis suggests that the subsurface, less saline water of the Arabian Sea are undergoing the most intense warming and saltening trends among the region’s upper oceanic water masses.

In the BoB, depth-wise distributions of upper oceanic water masses includes the Bay of Bengal Water (0–50 m), Mixed Zone (50–150 m), Indonesian Throughflow (200–500 m) and Indian Ocean Deep Water (below 500 m) as outlined in Table 1 and Fig. 4a)^54,59,60^. The isopycnals associated with both the BoB and Indonesian Throughflow (Fig. 4a) display deepening trends, whereas those linked to the Indian Ocean Deep Water are ascending (Fig. 4b). Distinct negative vertical temperature gradients are present, with a peak around 90 m, marking the most pronounced gradient among all regions (Fig. 4c). Consequently, the heave component of temperature changes displays a significant warming trend within the top 400 m (Fig. 5b) and a cooling pattern below 500 m. The spice component exhibits a cooling trend from 30 to 120 m peaking at roughly 60 m depth, followed by a steadily increasing warming trend below 120 m (Fig. 5b). The combined effects of spice and heave result in an isobaric warming trend below 100 m, with its pinnacle at about 150 m.

The vertical gradient of salinity transitions from positive to negative around the 75 m depth mark (Fig. 4d). In terms of salinity changes, the heave component is significant up to 80 m, manifesting freshening trends (Fig. 5i). The spice component for salinity changes delineates freshening trends above 120 m and saltening trends below (Fig. 5i). The total salinity change along the isobars exhibits a freshening pattern in the top 80 m, reaching an extreme value at the surface (~ − 0.023 psu/year), driven by both heave and spice components. In contrast, deeper regions witness changes primarily due to singular influence of warming spice, remaining relatively consistent throughout the depth (Fig. 5i).

In the upper layer of the west Equatorial Indian Ocean, rising isopycnals between ${\sigma }_{t}$ = 22.5–23.5 kg m^−3^ (Fig. 4b) are associated with the water masses of the Arabian Sea High Salinity Water (0–80 m). Conversely, deepening isopycnals ranging between ${\sigma }_{t}$ = 23.5–27.5 kg m^−3^ (Fig. 4b) correspond to the Indian Equatorial Water (100–550 m) and the Red Sea-Persian Gulf Intermediate Water (below 580 m) as detailed in Fig. 4a and Table 1^46,58,61^. This isopycnal deepening, combined with the observed negative vertical temperature gradient (Fig. 4c) results in a warming trend from the heave component between 60 and 500 m peaking around 120 m (Fig. 5c). While the spice component reveals warming trends across the entire depth, its dominant influence emerges below 300 m (Fig. 5c). Consequently, warming trends along isobars become evident, with a notable maximum around 120 m, and are primarily driven by the spice component below 400 m (Fig. 5c).

In this region, the salinity’s negative vertical gradient starts below 70 m (Fig. 4d). When combined with the deepening of isopycnals, it results in a subdued saltening trend from the heave component between 100 and 400 m (Fig. 5j). The spice component, which displays saltening trends consistently throughout depth and peaks at the surface, predominantly dictates the overall isobaric saltening trends in the upper ocean (Fig. 5j), with a maximum value at the surface of approximately 0.006 psu/year.

In the central Equatorial Indian Ocean region, the observed deepening of isopycnals (Fig. 4a,b) corresponds with the water masses of the Equatorial Upper surface (0–100 m) and subsurface (100–500 m) waters. Additionally, an increase is evident in the Red Sea-Persian Gulf Intermediate Water (below 580 m) as detailed in Table 1^55,61^. Consistent negative vertical temperature gradients are observed across the entire depth, peaking at around 95 m, signifying a layer of warm water atop colder water (Fig. 4c). As a result, the heave component reveals prominent warming trends up to 400 m, peaking at approximately 90 m. In contrast, the spice component exhibits significant warming trends starting below 250 m (Fig. 5d). The overall warming trends in temperature changes along isobars are predominately driven by the heave in the upper 250 m and by the spice below 400 m depth (Fig. 5d).

Regarding salinity, a positive vertical gradient is present up to 80 m, shifting to negative between 80 and 150 m (Fig. 4d). Consequently, the heave component displays a pronounced freshening trend above 80 m (Fig. 5k). Beyond 200 m depth, only the spice-related warming trend influences the isobaric salinity change (Fig. 5k).

In the eastern equatorial Indian Ocean, isopycnals deepening between 21.5 and 25.8 kg m^−3^ and 26.3–27.3 kg m^−3^, (Fig. 4b) correspond with the water masses of the BoB (0–50 m), Equatorial Surface (50–100 m) and subsurface Water (100–500 m), and Red Sea-Persian Gulf Intermediate Water (below 580 m) as detailed in Fig. 4a and Table 1^46,55,61^. Prominent negative vertical temperature gradients up to 700 m, peaking around 100 m, suggest layers of warm water above colder waters (Fig. 4c). Consequently, the heave component reveals warming trends primarily within the top 100 m, peaking near 90 m, and again below 200 m (Fig. 5e). The spice component, in contrast, displays significant warming trends starting just below 100 m (Fig. 5e). Therefore, overall temperature changes along isobars exhibit marked warming trends throughout the entire depth, most pronounced around 90 m, and predominantly influenced by the heave (Fig. 5e).

Conversely, for salinity, pronounced positive gradients peak at 50 m within the top 100 m (Fig. 4d). When coupled with the deepening of isopycnals, this results in freshening trends from the heave component in salinity changes (Fig. 5l). Below 100 m depth, the spice component demonstrates significant saltening trends, peaking around 120 m, which predominantly shape the overall isobaric salinity changes (Fig. 5l).

In the SCTR region, rising isopycnals between 22 and 22.8 kg m^−3^ (Fig. 4b) are linked to the less saline and warm surface water. In contrast, subsurface isopycnals ranging from 23 to 30 kg m^−3^, associated with the water masses of the Indian Equatorial (100–500 m) and Red Sea-Persian Gulf Intermediate Water (below 580 m) display deepening trends (Fig. 4a, Table 1)^55^. Both temperature and salinity exhibit distinct vertical gradients within the upper 70 m, peaking around 60 m, which confirm the presence of less saline and warmer water at the surface (Fig. 4c, 4d). The heave component of temperature changes reveals a warming trend throughout the upper layer, peaking at 80 m, but becomes statistically significant only below 150 m (Fig. 5f). In contrast, the spice component of temperature changes exhibits notable warming in the top 250 m, reaching its maximum at the surface (Fig. 5f). Consequently, total temperature changes along isobars present warming trends in the upper layer, peaking at 10 m, and predominantly adhere to the heave pattern, especially after the weakening influence of the spice component (Fig. 5f).

Regarding salinity, the spice component delineates a steadily diminishing saltening trend up to 300 m, whereas the heave’s influence is virtually non-existent in the upper layer (Fig. 5m). Thus, the overall salinity changes along isobars are chiefly governed by the spice, indicating saltening trends exclusively in the upper 300 m, and peaking at the surface (~ 0.015 psu/year).

In the SIO region, vertically averaged temperature and salinity do not show noteworthy trends in upper ocean (Fig. 3g,n). However, significant thermohaline changes are observed at several depths (Fig. 5g,n). Here is an observed uplift of isopycnals between 23 and 25.6 kg m^−3^, aligning with the Indonesian Upper Water (0–150 m). Conversely, isopycnals between 25.7 and 27 kg m^−3^ sink, associated with the Subtropical Surface Water (150–350 m) and Subantarctic Mode water (below 350 m) (Fig. 4a,b, Table 1)^55^. The negative vertical temperature gradients can be attributed to the layering of relatively warmer water over colder water (Fig. 4c). Nevertheless, the spice and heave components provide contrasting influences on the temperature changes along isobars (Fig. 5g). While the heave component presents cooling trends above 250 m depth and significant warming trends below, the spice component inversely displays significant warming trends above 250 m and cooling trends below, with pronounced extremes at the surface and around 450 m. Consequently, the aggregate isobaric temperature changes exhibit distinct warming trends between 80 and 300 m and cooling trends from 300 to 600 m (Fig. 5g).

Regarding salinity, the vertical gradient shifts from positive to negative around the 250 m mark (Fig. 4d). In this context, the heave component reveals a marked saltening trend beneath 250 m. However, the overarching salinity changes along isobars predominately reflect the influence of the spice component, resulting in saltening trends above 250 m and freshening trends below, peaking at the surface and around 400 m, respectively (Fig. 5n).

### Roles of three ventilation processes

The sea surface is predominantly influenced by buoyancy fluxes and wind. Both temperature and salinity in the ocean’s interior are conservative properties, meaning their alterations arise solely from advection and mixing processes. Neither the spice nor the heave components of temperature and salinity changes directly correlate to a specific process or atmospheric forcing. In this section, we dissect the trends of these components in temperature and salinity through three distinct ventilation processes: the pure warming, pure freshening, and pure heave process. Pure warming pertains to temperature fluctuations that occur without influencing salinity changes along isobars, essentially driven by the redistribution of surface heat via prevailing ocean circulation. Conversely, pure freshening encompasses salinity shifts without affecting temperature along isobars, reflecting either freshwater loss or addition at the surface. The pure heave process signifies the adiabatic elevation or depression of isopycnals without any spice-related alteration. Such movement is tied to thermohaline shifts influenced by various factors, including local or remote wind forcing, eddy activities, wave propagation or overarching large-scale gyre circulation.

**Figure 6:**
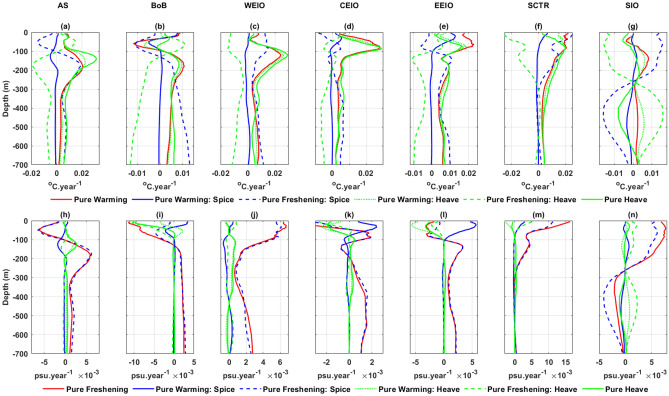
Contributions of pure warming, pure freshening and pure heave processes to the total (red), spice (blue) and heave (green) components of the temperature (top rows) and salinity trends (bottom rows) over the upper 700 m in the seven IO regions. Pure warming and pure freshening processes are shown as solid red lines; pure heave processes are represented by solid green lines; heave components induced by the pure warming process and pure freshening process are designated by dotted and dashed green lines, respectively; and spice components induced by the pure warming process and pure freshening process are denoted by solid and dashed blue lines, respectively.

In the AS, the pure warming process reveals warming trends along isobars within the upper strata, peaking around 200 m (Fig. 6a). Below a depth of 100 m, the warming spice emerges due to warming trends from the pure freshening process, countering minor cooling trends observed between 80 and 180 m under the pure warming process (Fig. 6a). Notable warming heave, evident below 100 m, results from an interplay of warming trends from the pure warming and heave processes, and cooling trends from the pure freshening process (Fig. 6a). The pure freshening process displays distinct freshening trends above 100 m and saltening trends below, with notable peaks at approximately 50 m and 170 m, respectively (Fig. 6h). Saltening spice, discernible below 100 m, is attributed to the saltening trends of the pure freshening process, contrasting with minor freshening trends between 80 and 180 m (Fig. 6h). A pronounced saltening heave in the subsurface arises from saltening trends influenced by both the pure warming and heave processes, while the freshening trends are steered by the pure freshening (Fig. 6h). Freshening spices within the top 100 m are exclusively tied to the pure freshening process (Fig. 6h).

In the BoB, the pure warming process exhibits cooling trends between 30 and 100 m and warming trends below this depth. Peaks for these trends are observed at approximately 60 m and 180 m, respectively (Fig. 6b). The pure freshening process demonstrates freshening trends above 120 m and saltening trends below this mark (Fig. 6i). Above 120 m, the cooling and freshening spice components arise from the combined effects of the pure warming and freshening processes (Fig. 6b,i). Conversely, below 120 m, warming and saltening spice are solely attributed to the pure freshening process (Fig. 6b,i). Heave-induced temperature fluctuations below 120 m are influenced by conflicting factors: warming trends from the pure warming and heave processes, and cooling trends from the pure freshening process (Fig. 6b). The freshening heave observed within the top 100 m predominantly stems from the pure freshening and heave processes (Fig. 6i).

In the WEIO, the pure warming processes displays a warming trend along isobars, peaking at approximately 120 m (Fig. 6c). The pure freshening process reveals saltening trends along isobars, reaching a maximum at the surface (Fig. 6j). In this context, both the warming and saltening spices are exclusively attributed to the pure freshening process (Fig. 6c,j). Nonetheless, the combined effects of warming trends from the pure warming and heave processes, and the cooling trend from the pure freshening process, lead to a subdued warming heave compared to the change in spice (Fig. 6c).

In the CEIO, the pure warming process reveals a warming trend along isobars, peaking at approximately 90 m (Fig. 6d). The pure freshening process demonstrates saltening trends along isobars between 40 and 120 m, and again below 150 m (Fig. 6k). Below 200 m, the warming and saltening spices can be solely attributed to the trends observed in the pure freshening process (Fig. 6d,k). The warming heave arises from the interplay between warming trends from the pure warming and heave processes, counteracted by cooling trends from the pure freshening process (Fig. 6d). Notably, a significant freshening heave in the top 80 m results from the cumulative freshening trends driven by the pure warming, heave and freshening processes (Fig. 6k).

In the EEIO region, the pure warming displays warming trends along isobars, peaking at approximately 90 m (Fig. 6e). The pure freshening reveals freshening trends above 100 m and saltening trends below it (Fig. 6l). In the upper 100 m, the warming spice results from the countering effects of a warming trend from pure warming and a cooling trend from the pure freshening (Fig. 6e). Below this depth, the warming and saltening spices are solely attributed to the pure freshening process (Fig. 6e,l). The warming and freshening heave in the top 100 m arises from combined effects of trends from pure warming, heave and freshening processes (Fig. 6e,l). However, below 100 m, the warming heave stems from contrasting influences: warming trends from pure warming and heave processes counteracted by cooling trends from pure freshening (Fig. 6e).

In the SCTR region, the pure warming reveals warming trends along isobars, peaking within the top 80 m (Fig. 6f). The pure freshening process exhibits pronounced saltening trends up to 300 m, most prominently at the surface (Fig. 6m). Here, the warming (and saltening) spice predominantly stems from the pure freshening process, with a minor contribution from the pure warming process in the uppermost 80 m (Fig. 6f,m). Warming trends in the heave below 50 m arise from opposing influences: warming trends from both the pure warming and heave processes are counteracted by a cooling trend from the pure freshening process (Fig. 6f).

In the SIO region, the pure warming exhibits a cooling trend in the top 80 m and a warming trend thereafter, with pronounced values between 80 and 250 m (Fig. 6g). The pure freshening reveals a saltening trend up to 250 m and freshening beyond that depth (Fig. 6n). The spice-related warming (saltening) and cooling (freshening) trends above and below 250 m predominantly arise from the pure freshening (Fig. 6g,n). Concurrently, the cooling heave within the top 250 m stems from cooling tendencies due to the pure freshening and pure heave above 100 m, and warming tendencies between 100 and 250 m from the pure warming (Fig. 6g). Beyond 250 m, the warming heave is shaped by opposing influences: warming from the pure freshening and pure warming, and cooling from the pure heave (Fig. 6g).

## Discussion

This study examines the upper oceanic thermohaline changes within the tropical Indian Ocean from 2003 to 2019 using Argo TS profiles. It focuses on seven distinct regions of the Indian Ocean, chosen for their notable characteristics. Within these regions, water mass distributions provide context for the upper oceanic isopycnals. A warming trend in the average upper oceanic temperature anomaly is noted across all Indian Ocean regions, excluding the southern Indian Ocean. On the other hand, the average upper oceanic salinity anomaly indicates a saltier trend in the AS, WEIO, CEIO, EEIO, and SCTR regions (Fig. 1). Notably, warming trends are evident at various depths in all regions, excluding the SIO and the upper 100 m of the BoB. The study underscores three key patterns consistent across the seven regions: (1) Below 300 m, the heave component is minimal, with the spice component prevailing; (2) Under 300 m, the freshening process primarily drives observed trends within the spice component; (3) The spice component primarily influences salinity changes along isobars across all regions.

Here are some region-specific highlights:

- In the Arabian Sea, below 100 m, there's a notable warming and saltier trend. The Subsurface Salinity Minimum Water exhibits pronounced thermohaline shifts, peaking at around 150 m, more so than in the Persian Gulf and Red Sea. These trends in the Subsurface Salinity Minimum Water arise from the combined influence of spice component’s changes via pure freshening and the heave component's effects through pure warming and pure heave.
- In the Bay of Bengal (BoB), the most pronounced cooling and warming trends are seen at around 60 m and 150 m, respectively. The former is linked to the mixing zone's interaction with Bay of Bengal Water, while the latter correlates with the Indonesian Throughflow. The Bay of Bengal Water primarily freshens due to the combined effects of pure freshening and heave. Conversely, both the Indonesian Throughflow and the Indian Ocean Deep Water consistently warm and become saltier below 200 m.
- In the western equatorial Indian Ocean, the most pronounced warming trend occurs around 120 m, aligning with the Indian Equatorial water characteristics. The saltier trend observed, especially in the surface layer, results from the spice influence under the pure freshening mechanism.
- In the central equatorial Indian Ocean, the dominant warming trend appears around 90 m. This significant warming within the top 100 m stems from shifts in the heave component influenced by pure warming and heave mechanisms. Below 200 m, both the subsurface equatorial water and the Red Sea-Persian Gulf Intermediate water exhibit warming and saltier trends, attributed to the influence of spice components under the pure freshening mechanism.
- In the east equatorial Indian Ocean, the upper 100 m displays a warming trend influenced by the spice component's pure warming effect and the heave component's dual pure warming and heave effects. Deeper than 100 m, both the Indian Equatorial and Red Sea-Persian Gulf Intermediate waters show warming and saltier tendencies, driven by the spice component through the pure freshening mechanism.
- In the SCTR region, the combined effects of pure warming and freshening contribute to spice component and amplify the warming and saltier trends, peaking in surface waters. While both the Indian Equatorial and Red Sea-Persian Gulf Intermediate waters demonstrate warming as depth increases, neither exhibits a saltier trend below 300 m.
- In the Southern Indian Ocean (SIO), the spice component primarily drives thermohaline shifts under the pure freshening mechanism. Between 80 and 300 m, distinct warming and cooling trends are evident. Specifically, saltier trends are noticeable above 250 m, while freshening trends dominate below this depth. Notably, the advected mode water exhibits significant freshening and cooling trends.

These findings would offer insights into recent shifts in oxygen-depleted water distributions^22^ and related ecosystems^21^. They can also shed light on the increasing frequency and intensity of marine heatwaves^62^ and tropical cyclones^63^ in the Indian Ocean. The results presented here on the elements of thermohaline patterns may be relevant to the recent increases in sea level observed in the Indian Ocean^64,65^, which warrants future investigation.

## Methods

### Data

This study utilized 17 years (2003–2019) of Argo dataset^66^, which provides high-resolution temperature-salinity profiles up to 2000 m. The accuracies of temperature, salinity and pressure measurements in the Argo profile are $\pm$ 0.002 $^\circ{\rm C}$, $\pm$ 0.01 psu, and $\pm$ 2.4 dbar, respectively (https://argo.ucsd.edu/). Before utilizing this data, we conducted quality control tests based on recommendations from the Argo quality control manual (version 3.3)^67^. Subsequent tests were conducted to eliminate values deviating from statistical norms. In 2003, the number of quality-controlled Argo profiles in the IO region (spanning 30° S–30° N and 20° E–120° E) was 3199, which increased to 12,619 by 2019. Notably, the quality of Argo observations has enhanced; rejected datasets post-quality control dropped from 35% in 2003 to 7.6% in 2016. However, this rate later rose to 20%. This surge is quality Argo profiles has bridged the data observation gaps. While temperature and salinity profiles from Argos are typically available at ~ 2 m intervals, the depths of individual Argo floats differ. Consequently, we interpolated these profiles to standard levels. The top 100 m consists of 20 levels starting at 5 m. Following this, intervals expand: 10 m up to 200 m, 20 m up to 400 m, 50 m up to 1000 m, and 100 m between 1000 and 2000 m. We also calculated the monthly mean temperature and salinity profiles yearly for each study region, averaging all the quality-controlled profiles available for each month and region. Figure 1 displays the total number of these quality-controlled Argo profiles for each region from 2003 to 2019. For our time series analysis, we opted against using filters.

We compared and correlated Argo’s near surface temperature (NST) and salinity (NSS) at 5 m depth with several datasets. These include the monthly NOAA Optimum Interpolation (OI) SST version 2 dataset^68^, which has a spatial resolution 1 $^\circ \times$ 1 $^\circ$, and the monthly Level 3 mapped Aquarius/SAC-D version 5.0 sea surface salinity (SSS) data^69^ from September 2011 to May 2015 with a resolution 0.5 $^\circ \times$ 0.5 $^\circ$. We also considered the SMAP (Soil Moisture Active Passive) SSS version 4.0 data^70^ available from April 2015 to December 2019, with a resolution of 0.25 $^\circ \times$ 0.25 $^\circ$. However, we did not include the Soil Moisture and Ocean Salinity (SMOS) Level 4 SSS data (https://data.marine.copernicus.eu/product/MULTIOBS_GLO_PHY_SSS_L4_MY_015_015/description), which started in December 2010 and offers weekly updates at a 1 $^\circ \times$ 1 $^\circ$ spatial resolution. The exclusion was due to its insufficient spatial coverage, especially in the northern Indian Ocean^71^.

### Formulae

#### Spice-heave decomposition of total change

**Figure 7:**
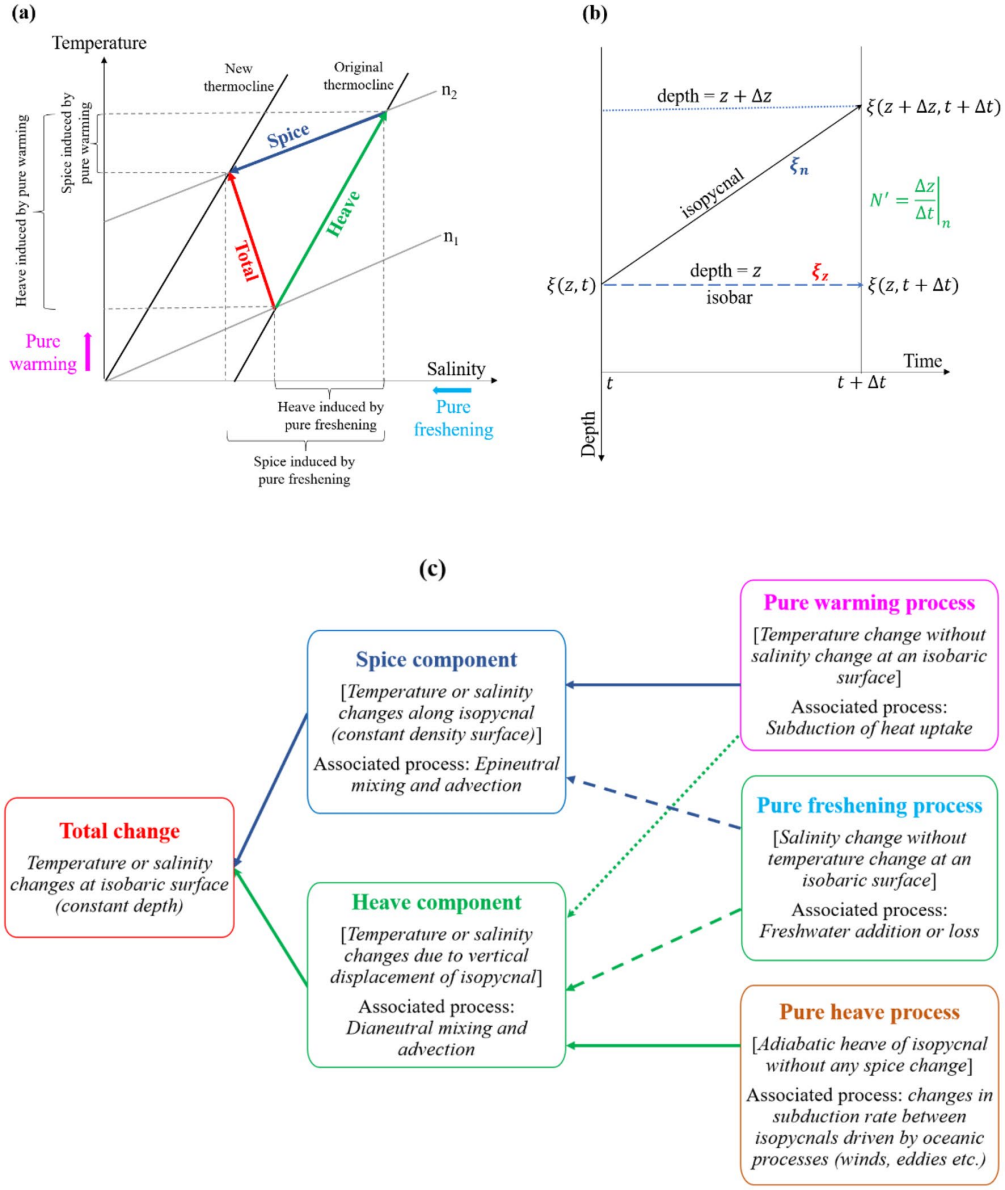
(**a**) In a T–S space, schematic diagram illustrates the total (red arrow), spice (blue arrow) and heave (green arrow) components of water mass property change, and the contribution of pure warming and pure freshening to spice- and heave-related changes^13,27,30^; (**b**) The sketch exhibits the changes of water mass property along the isobaric and isopycnal surfaces with the time span; (**c**) Hierarchy of the components and associated processes responsible for the changes water mass properties in water column.

To examine variations in temperature and salinity across specific sub-regions and associate them with water masses, we adopted the methodology introduced by Bindoff and Mcdougall (1994)^27^. This approach, which has been further modified and utilized by numerous researchers^30,36^, proposed that changes in water mass properties, such as temperature (T) or salinity (S) at a fixed depth (on an isobar) can be broken down into two components (as illustrated in Fig. 7a): (i) changes in water mass composition along a neutral density surface, i.e., isopycnal, and (ii) modifications in water mass composition due to the vertical movement of the isopycnal. Häkkinen et al. (2016) later labeled the breakdown of the overall shift (change along isobar, illustrated by red arrow in Fig. 7a) as (i) the spice component (changes along isopycnal, represented by the blue arrow in Fig. 7a) and (ii) the heave component (changes due to a vertical shift of isopycnal, shown by the green arrow in Fig. 7a). These two elements contributing to the comprehensive water mass property variations are calculated following a specific decomposition equation ^27^. A detailed derivation of this equation is provided below. This type of decomposition has been applied in several studies^13,27–40,42–44^ to elucidate thermohaline changes in the water columns of the global ocean basins.

Suppose, at a certain depth z and time t, the water mass property is $\xi =\xi (z,t)$. At the same depth (along isobar) after a time span of $\Delta t$ (Fig. 7b, blue dashed line with arrow), the property becomes $\xi (z,t+\Delta t)$. Applying the first order approximation of the Taylor series, we get

$$ \xi \left( {z,t + \Delta t} \right) \approx \xi \left( {z,t} \right) + \Delta t\frac{\partial \xi }{{\partial t}} $$

1 $$ \Rightarrow \frac{\partial \xi }{{\partial t}} = \frac{{\xi \left( {z,t + \Delta t} \right) - \xi \left( {z,t} \right)}}{\Delta t} = \xi_{z}^{\prime} $$

However, along the isopycnal after time span of $\Delta t$ (Fig. 7b, solid black line with arrow), the property will be $\xi (z+\Delta z,t+\Delta t)$. Using the first order approximation of the Taylor series, we obtain

$$ \xi \left( {z + \Delta z,t + \Delta t} \right) \approx \xi \left( {z,t} \right) + \Delta z\frac{\partial \xi }{{\partial z}} + \Delta t\frac{\partial \xi }{{\partial t}} $$

2 $$ \Rightarrow \frac{\partial \xi }{{\partial t}} = \frac{{\xi \left( {z + \Delta z,t + \Delta t} \right) - \xi \left( {z,t} \right)}}{\Delta t} - \frac{\Delta z}{{\Delta t}}\frac{\partial \xi }{{\partial z}} = \xi_{n}^{\prime} - N^{\prime}\xi_{z} $$

From Eqs. (1) and (2), it can be written that

3 $$ \underbrace {{\xi_{z}^{\prime} }}_{{\begin{array}{*{20}c} {total} \\ {change} \\ \end{array} }} = \underbrace {{\xi_{n}^{\prime} }}_{{\begin{array}{*{20}c} {spice} \\ {component} \\ \end{array} }} - \underbrace {{N^{\prime}\xi_{z} }}_{{\begin{array}{*{20}c} {heave} \\ {component} \\ \end{array} }} $$

where the prime symbol in the superscript denotes the temporal changes $\left(\frac{d}{dt}\right)$; z and n in the subscript indicate along pressure (isobaric) and neutral density (isopycnal) surfaces, respectively; $N^{\prime}$ represents the rate of change of isopycnal depth $\left({\left.\frac{dz}{dt}\right|}_{n}\right)$; and ${\xi }_{z}$ is the vertical gradient of water mass property $\left(\frac{\partial \xi }{\partial z}\right)$ considered constant with time. The discrepancy between the overall shift and the combined total of both components, as represented in Eq. (3) yields a residual. The first term on the right-hand side of Eq. (3) termed the change in spice^13^, pertains to the variability in heat and freshwater flux within the water mass formation area^39^. It reflects epineutral mixing or advection^35^. Conversely, the second term on the right-hand side of Eq. (3), labeled as the change in heave^13^, arises either from adiabatic processes (such as wind forcing, eddy activity, and low-frequency Rossby waves) or diabatic flux divergence (like downward diffusion of surface heating or freshening, and changes in the water mass renewal rate).

#### Ventilation processes

The spice and heave components of both temperature and salinity shifts, encompass more than just distinct processes or specific atmospheric influences. In a steady-state ocean, epineutral (along the isopycnal) and dineutral (across the isopycnal) mixing and advections are crucial for conserving volume on and between isopycnals. Such a steady-state ocean assumes consistent surface boundary influences from the wind, heat, and freshwater fluxes. Yet, changes in these surface forces each contribute notably to thermohaline changes along isobars and isopycnals by either redistributing inherent properties within the water column or by altering the circulation. Bindoff and McDougall (1994)^27^ labeled the impact of the changes in these three boundary conditions as pure warming (${T}_{z}^{\prime}>0, {S}_{z}^{\prime}=0$), pure freshening (${T}_{z}^{\prime}=0, {S}_{z}^{\prime}<0$) and pure heave processes (${T}_{n}^{\prime}=0, {S}_{n}^{\prime}=0$)^42^. They proposed a model in density units, which analyzed these three ventilation processes individually, investigating their ties to thermohaline shifts. The expression of the model is as follows:

4 $$\frac{{\rho }^{-1}{\rho }_{z}^{\prime}}{{R}_{\rho }-1}\left[\begin{array}{ccc}-({R}_{\rho }-1)& 0& {-R}_{\rho }\\ 1& {R}_{\rho }& 0\\ {R}_{\rho }& {R}_{\rho }& {R}_{\rho }\\ 0& {R}_{\rho }-1& -1\\ 1& {R}_{\rho }& 0\\ 1& 1& 1\end{array}\right]\left[\begin{array}{c}{A}_{w}\\ {A}_{f}\\ {A}_{h}\end{array}\right]=\left[\begin{array}{c}\alpha {T}_{z}^{\prime}\\ \alpha {T}_{n}^{\prime}\\ \alpha {{N}^{\prime}T}_{z}\\ \beta {S}_{z}^{\prime}\\ \beta {S}_{n}^{\prime}\\ \beta {{N}^{\prime}S}_{z}\end{array}\right]$$

where the unknown coefficients A_w_, A_f_, and A_h_ represent the relative strengths of the three processes: warming, freshening and heaving, represented by w, f, and h, respectively. The symbol $\rho$ signifies the average seawater density, while ${\rho }_{z}^{\prime}$ indicates the density variation along isobars. The stability ratio is given by ${R}_{\rho } =\alpha {T}_{z}/\beta {S}_{z}$, where $\alpha$ and $\beta$ are the thermal expansion and haline contraction coefficients of seawater, respectively. While Eq. (4) poses challenges due to its ill-posed nature, we address this linear system through the singular value decomposition (SVD) technique. This approach offers least-squares solutions that are optimally concise^30^. For this research, we exclusively incorporate linear trend values that meet the 90% significance threshold (with a *p*-value $\le$ 0.1), discarding the null hypothesis. The roles of pure warming, freshening, and heaving in the spice and heave elements are depicted in the provided schematics (Fig. 7a–c).

## Data Availability

Temperature and salinity profiles from the Argo dataset^66^ are freely available from https://data-argo.ifremer.fr/geo/indian_ocean/. The OISST^68^ version 2 is obtained from https://psl.noaa.gov/data/gridded/data.noaa.oisst.v2.html. Monthly Level 3 Aquarius/SAC-D SSS^69^ version 5.0 during September 2011–May 2015 are obtained from https://podaac.jpl.nasa.gov/dataset/AQUARIUS_L3_SSS_SMI_MONTHLY_V5. The SMAP SSS^70^ version 4.0 April 2015–December 2019 is taken from https://podaac.jpl.nasa.gov/dataset/SMAP_JPL_L3_SSS_CAP_MONTHLY_V5 data.
